# Characterization of CTNND2-related neurodevelopmental disease, phenotype-genotype spectrum and WNT dynamics in early neurogenesis

**DOI:** 10.21203/rs.3.rs-8224288/v1

**Published:** 2025-12-30

**Authors:** Mansoureh Shahsavani, Josephine Wincent, Ricarda Reiter, Andrea Soltysova, Jakob Schuy, Hafdis T. Helgadottir, Jesper Eisfeldt, Marlene EK, Andrej Ficek, Lotta Druschke, Katarina Kusikova, Tzung-Chien Hsieh, Aron Krichhoff, Peter Krawitz, Jing-Mei Li, Gerald Webersinke, Svetlana Gorokhova, Chantal Missirian, Florence Riccardi, Lisa Pavinato, Alfredo Brusco, Giorgia Mandrile, Slavica Trajkova, Francesco Pintus, Biljana Gagachovska, Quinten Waisfisz, Annet van Hagen, Emma Bedoukian, Kosuke Izumi, Leslie Granger, Andrea Petersen, Renske Oegema, Manon Huibers, Florence Demurger, Elise Brischoux-Boucher, Sophie Julia, Guillaume Banneau, M. Jesus Zavala, Catalina Lagos, Gabriela M. Repetto, Guillaume Jouret, Catherine Kentros, Mythily Ganapathi, Wendy K. Chung, Halie May, Susan M. Hiatt, Whitley V. Kelley, Alisa Förster, Lisa Olfe, Amelle Shillington, Benjamin Dauriat, Sandra Mercier, Benjamin Cogné, Camille Engel, Eric Dahlen, Georg Rosenberger, Thomas Sauvigny, Hamza Hadj Abdallah, Thomas Courtin, Asbjørg Stray-Pedersen, John A. Bernat, Vitoria K Paolillo, Florencia Del Viso, Joseph T Alaimo, Isabelle Thiffault, Emily G Farrow, Ana S.A. Cohen, Serge Weis, Hans-Christoph Duba, Ann Nordgren, Anna Falk, Denisa Weis, Anna Lindstrand

**Affiliations:** Karolinska Institutet; Karolinska Institutet; Johannes Kepler University of Linz; Comenius University Bratislava; Karolinska Institutet; Karolinska Institutet; Karolinska University Hospital; Karolinska Institutet; Comenius University Bratislava; Karolinska Institutet; Comenius University and National Institute of Childreńs Diseases; University Hospital Bonn, Rheinische Friedrich-Wilhelms-Universität Bonn; University Hospital Bonn, Rheinische Friedrich-Wilhelms-Universität Bonn; University Hospital Bonn, Rheinische Friedrich-Wilhelms-Universität Bonn; University Hospital Bonn, Rheinische Friedrich-Wilhelms-Universität Bonn; Ordensklinikum Linz Barmherzige Schwestern; La Timone Children Hospital, AP-HM; La Timone Children Hospital, AP-HM; Aix-Marseille University; Bellinzona Institutes of Science (BIOS+); Rita Levi-Montalcini, University of Torino; San Luigi University Hospital, University of Torino; Rita Levi-Montalcini, University of Torino; University of Turin; University Clinic of Psychiatry, Faculty of Medicine, Ss. Cyril and Methodius University; Amsterdam UMC, Vrije Universiteit Amsterdam; Amsterdam UMC, Vrije Universiteit Amsterdam; Children’s Hospital of Philadelphia; Children’s Hospital of Philadelphia; University Medical Center Utrecht; University Medical Center Utrecht; University Medical Center Utrecht; University Medical Center Utrecht; Centre hospitalier Bretagne Atlantique; Université Marie et Louis Pasteur; CHU Purpan; CHU Purpan; Universidad del Desarrollo; Universidad del Desarrollo; Universidad del Desarrollo; Laboratoire National de Santé; Columbia University; Columbia University Irving Medical Center; Harvard Medical School; Columbia University Irving Medical Center; Huntsville, AL; Huntsville, AL; Hannover Medical School; Hannover Medical School; University of Cincinnati College of Medicine; Hôpital Mère-Enfant; Centre Hospitalier Universitaire de Nantes; Centre Hospitalier Universitaire de Nantes; Université Bourgogne Franche-Comté; Centre Hospitalier Universitaire de Besançon; University Medical Center Hamburg-Eppendorf; University Medical Center Hamburg-Eppendorf; Hôpital Necker-Enfants Malades; Hôpital Necker-Enfants Malades; Oslo University Hospital; University of Iowa Health Care; Children’s Mercy Hospital; Children’s Mercy Hospital; Children’s Mercy Hospital; Children’s Mercy Hospital; Children’s Mercy Hospital; Children’s Mercy Hospital; Johannes Kepler University of Linz; Johannes Kepler University of Linz; Karolinska Institutet; Karolinska Institutet; Johannes Kepler University of Linz; Karolinska Institutet

**Keywords:** CTNND2, δ-catenin, WNT signaling, neurodevelopmental disorder, early neurogenesis, induced pluripotent stem cells, CRISPR-Cas9

## Abstract

**Background:**

Heterozygous variants in *CTNND2*, encoding the brain-specific protein δ-catenin, are associated with a broad spectrum of neurodevelopmental disorders, including dyslexia, attention deficit hyperactivity disorder, intellectual disability, and autism. Despite its clinical significance, the full phenotypic spectrum of *CTNND2*-associated disorders and the neurodevelopmental role of δ-catenin, a key component of the cadherin-catenin cell adhesion complex, remain poorly defined.

**Methods:**

Through international collaboration, we assembled the phenotypic and molecular information for 57 individuals, 42 previously unpublished, carrying heterozygous *CTNND2* variants. All individuals were evaluated by local clinicians, and the variants were identified through exome or genome sequencing, clinical microarray, or karyotyping. To investigate the effects of δ-catenin loss on early neurogenesis, we performed neural differentiation and transcriptomic profiling in three patient-derived neural stem cell lines and three CRISPR-Cas9-generated *CTNND2* knockout lines. In one patient-derived line, we further analyzed cerebral organoid development and performed pathway modulation to assess phenotypic rescue.

**Results:**

The 41 *CTNND2* variants included 12 previously reported loss-of-function- and one missense variant, and 28 novel variants comprising 10 missense and 18 predicted loss-of-function changes. Eight of the novel variants occurred *de novo*, and 12 were inherited from a parent with a neurodevelopmental phenotype. The most common clinical features were developmental delay (90%), intellectual disability (74%), and behavioral abnormalities (79%). Functional studies revealed impaired early neurogenesis in one patient-derived line, characterized by aberrant neural rosette formation. Transcriptome analysis showed dysregulated WNT signaling, and partial rescue of these defects was achieved by modulating the WNT pathway, highlighting δ-catenin’s role in early neural development.

**Conclusions:**

This study defines the clinical symptoms of *CTNND2*-related neurodevelopmental disorders, outlining a recognizable yet variable phenotype that overlaps with other forms of intellectual disability and autism. Our findings provide preliminary evidence of genotype–phenotype correlations and highlight δ-catenin’s critical role in modulating WNT signaling during early neural development. These insights advance our understanding of *CTNND2*-associated disorders and support the importance of mechanistic studies to inform personalized diagnostics and therapies.

## BACKGROUND

Loss of function variants (LOF) affecting *CTNND2*, located on the short arm of chromosome 5, have been linked to various neurodevelopmental disorders (NDD) and psychiatric conditions such as intellectual disability (ID) with or without dyslexia-like learning difficulties (Belcaro et al., 2015; Hofmeister et al., 2015), autism spectrum disorder (ASD) (Miller et al., 2020; Turner et al., 2015), attention deficit hyperactivity disorder (ADHD) (Adegbola et al., 2020), and schizophrenia (Chen et al., 2023). Furthermore, a recent report details a consanguineous family in which three siblings harbored biallelic deletions at 5p15.2 encompassing 19 out of 22 exons of *CTNND2*. These individuals exhibited severe NDD, clinically more severe than heterozygous family members (Pauly et al., 2024).

*CTNND2* encodes the cytosolic protein delta 2-catenin (δ-catenin) and, together with β-catenin, belongs to the catenin superfamily. It is part of the cadherin-catenin cell adhesion complex involved in the canonical WNT/LEF-1-mediated pathway, activating respective gene transcription (H. Kim et al., 2012; Zhang et al., 2025). Previous studies in mice have shown that absence of CTNND2 results in severe learning deficits, synaptic plasticity impairments, and alterations in synaptic composition (Israely et al., 2004) as well as dendritic architecture and function (Arikkath et al., 2008, 2009; H. Kim, Han, et al., 2008; H. Kim, Oh, et al., 2008; K. Kim et al., 2002; Matter et al., 2009). Elevating *Ctnnd2* expression in mice resulted in heightened sociability and decreased anxiety while homozygous *Ctnnd2* knockout mice exhibited spatial learning deficits and aberrant fear conditioning (Ryu et al., 2019). More recently, using knockdown and knockout zebrafish models for *CTNND2*, we showed abnormal neuron migration and altered gene expression in the forebrain as well as increased swimming activity in both larval and adult stages (Hofmeister et al., 2015; Vaz et al., 2023).

Here, we detail both the clinical spectrum of *CTNND2*-related neurodevelopmental disease and its impact on neurogenesis. We report phenotype and genotype data from 57 individuals (including 15 previously published) with heterozygous *CTNND2* variants, collected through an international collaboration. Using induced pluripotent stem cell-derived neural cells from patients and CRISPR-Cas9-generated *CTNND2* knockout lines, we assessed effects on neural development. The findings define a distinct but variable condition and highlight dysregulation of WNT signaling dynamics as a key mediator of early neurogenesis abnormalities.

## METHODS

### Participants

We collected genotype and phenotype information for 57 individuals with heterozygous variants in *CTNND2* (Additional File 1: Table S1). Fifteen individuals had been previously reported (Sanders et al., 2011; Asadollahi et al., 2014; Belcaro et al., 2015; Hofmeister et al., 2015; Adegbola et al., 2020; Pauly et al., 2024; Ambrose et al., 2025;). The previously unreported individuals (n = 42) were identified through GeneMatcher (http://genematcher.org/) (Sobreira et al., 2015). Clinical information, including developmental history, cognition, neurological manifestations, behavioral features and dysmorphology, was reviewed by local clinicians. Frequencies of clinical symptoms are calculated based on the number of affected vs. assessed individuals. Variants in *CTNND2* classified as pathogenic or likely pathogenic (ACMG classes 5 and 4), as well as missense variants with a REVEL score > 0.4 and a gnomAD v2.1.1 allele frequency < 0.000009, were included.

### GestaltMatcher facial analysis

Facial photos from 15 individuals with *CTNND2* variants (N_7–12, N_17, N_17a, N_19, N_23, N_25, N_26, N_32, N_43, and N_58) were analyzed using the GestaltMatcher framework (Hsieh et al., 2022; Mak et al., 2025). Facial similarity was quantified by averaging pairwise cosine distances across 12 embeddings per image, generated using an ensemble model with test-time augmentation (Hustinx et al., 2022). Intra-cohort similarity was assessed by comparing their mean pairwise distance within the *CTNND2* group to reference distributions from the GestaltMatcher Database (Lesmann et al., 2024), which includes known syndromes and randomly sampled cohorts. Pairwise comparisons were also performed to evaluate facial similarity at the individual level. An average *CTNND2* facial image was created as a composite photo of all 15 individuals. Clinical feature prevalence is summarized in Additional File 1: Table S1, and detailed methods are provided in the supplementary files (Additional File 2: Supplementary methods).

### Identification and evaluation of genetic variants

*CTNND2* variants were identified through karyotyping, clinical microarray analysis, or exome and genome sequencing performed in diagnostic or research settings. Segregation analysis was performed when possible (n = 20). All identified variants were classified according to the American College of Medical Genetics and Genomics (ACMG) guidelines (Richards et al., 2015) (Additional File 1: Table S1). The Genome Aggregation Database (gnomAD v.2.1.1; https://gnomad.broadinstitute.org/) was used to assess the presence of these variants in control populations. Variant coordinates are based on the *CTNND2* transcript NM_001332.4 and the GRCh37/hg19 reference genome.

### Generation of iPSC, NESC, and knockout lines

Skin biopsies were obtained from three healthy controls (C#1, C#2, and C#3) and three individuals with pathogenic *CTNND2* variants. These included a 28-year-old female (N_48 hereafter called P#1) and her mother (N_49 - P#2), both carriers of a balanced chromosomal translocation disrupting *CTNND2*, and a four-year-old male (N_7 - P#3) with a 143 kb heterozygous deletion spanning exons 4–9 of *CTNND2*. Fibroblasts were cultured according to standard protocols and induced pluripotent stem cell (iPSC) lines were generated at the Karolinska Institutet iPS Core facility (Karolinska Institutet, Stockholm, Sweden) as previously described (Kele et al., 2016). Details of all cell lines used are provided in Additional File 1: Table S2.

To ensure genetic integrity, iPSC lines underwent standard G-banding karyotyping, SNP-array (Illumina Infinium Global Screening Array-24 BeadChip), and optical genome mapping (Bionano Saphyr). No significant chromosomal abnormalities, pathogenic copy number variants, or balanced structural rearrangements were detected, confirming that the lines remained genetically stable during reprogramming. Finally, the presence of the translocation was confirmed by breakpoint junction PCR.

*CTNND2* knockout (KO) lines were generated with CRISPR/Cas9 technology and validated at the Karolinska Genome Engineering (KGE) core and the KI iPSCore facilities utilizing C#1 as the parental line. Three clones were selected, characterized, and used for the study: KO#1 was heterozygous for the deletion, while KO#2 and KO#3 were homozygous (Additional File 3_Fig S1 A, B). Neural epithelial stem cells (NESCs) were derived from the nine iPSC lines (Additional File 1: Table S2) as previously described (Calvo-Garrido et al., 2021). Detailed protocols are provided in the supplementary files (Additional File 2: Supplementary methods).

### Generation of cerebral organoids and budding capacity

Cerebral organoids were generated using a modified version of the protocol by Lancaster and Knoblich (Lancaster & Knoblich, 2014), consisting of four main steps: embryoid body (EB) formation, neural induction, expansion, and maturation. Details are provided in the supplementary files (Additional File 2: Supplementary methods).

Embryoid bodies and organoids were imaged daily until day 15, and every other day from day 15 to day 30, with over 60 organoids analyzed per cell line. ImageJ v1.53S software was used for manual outlining of perimeters and bud counting. Quantitative parameters included organoid area, perimeter, and circularity ($4\pi \frac{\text{area}}{{\text{perimeter}}^{2}}$). Budding capacity was assessed by categorizing organoids into binary groups based on the presence or absence of buds and calculating the bud-per-organoid ratio. Statistical analysis of budding capacity was performed using a generalized linear model with binomial link function, assessing the effects of days in culture and cell line differences (C#1 vs P#1) on budding rates.

### Immunofluorescence staining and quantitative real-time PCR

Both NESCs and cerebral organoids were analyzed by immunofluorescence staining and quantitative real-time PCR (qPCR). For immunofluorescence, fixed samples were stained with antibodies against CTNND2 and key neural markers. Total RNA was extracted from NESCs and organoids, and cDNA was synthesized for qPCR analysis of *CTNND2* and selected target genes. Primer specificity and amplification efficiency were rigorously validated. Gene expression levels were normalized to housekeeping genes (*GAPDH* and *ACTB*) and compared to control cell lines. Detailed procedures, antibody information (Additional File 1: Table S3), and primer sequences (Additional File 1: Table S4) are provided in Additional File 2: Supplementary methods.

### Transcriptome analysis

Total RNA was extracted in triplicate from 2D-cultured cells at day 12/P0 and P6 using the RNeasy Mini Kit on the QIAcube Connect robot (QIAGEN). Bulk RNA-Seq was performed using the Illumina TruSeq Stranded mRNA library preparation protocol on a NovaSeq6000 platform (National Genomics Infrastructure, SciLifeLab, Stockholm). Raw sequencing reads were processed with an in-house pipeline (Ewels et al., 2020) nf-core/rnaseq (github.com/nf-core/rnaseq); which included quality control, data cleaning, read alignment to the GRCh38 reference genome was done with STAR (Dobin et al., 2013) and transcript quantification with Salmon (Patro et al., 2017).

Differential gene expression analysis was performed with DeSeq2 (Love et al., 2014), applying a false discovery rate (FDR) threshold of 1% and a 25%-fold-change cutoff. Principal component analysis (PCA) was used to assess sample clustering. Pathway enrichment was performed using the PANTHER database (Mi et al., 2005) and WNT pathway genes were annotated based on KEGG pathway (Kanehisa et al., 2016). Full details are provided in Additional File 2: Supplementary methods.

## RESULTS

### Cohort characteristics

We report 42 novel individuals from 29 pedigrees diagnosed with heterozygous variants in *CTNND2*. The novel cohort includes 17 males and 15 females, with a mean age of 15 years (range: 1 day to 49 years). Combined with 15 previously published cases, the total cohort includes 57 individuals: 32 male, 23 females and 2 with unknown sex. The cohort spans both pediatric cases (70%) and adults (21%) with age information unavailable for 9% of individuals. Clinical symptoms at the cohort level are summarized in Table 1 (frequencies are based on the number of affected vs. assessed individuals). The detailed clinical manifestations are listed in Additional file 1: Table S1.

### CTNND2 genotype spectrum CTNND2 genotype spectrum

**Figure 1:**
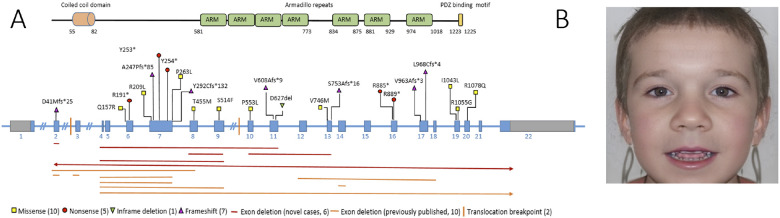
**A.** Schematic representation of δ-catenin (top) and the CTNND2 gene (bottom). Genetic variants are indicated at their respective positions on the gene: yellow squares represent missense variants, red circles nonsense variants, a green triangle an in-frame deletion, and a purple triangle a frameshift variant. Translocation breakpoints are shown as yellow vertical lines. Exon deletions are depicted as horizontal lines in red (new cases) and orange (previously published cases). **B.** Composite facial image of individuals with CTNND2-related disorders generated using GestaltMatcher photorealistic synthetic portrait (Kirchhoff et al., 2025).

In the 42 novel individuals, we identified 28 distinct *CTNND2* variants, classified as either LOF (n = 18, 6 large deletions, 5 nonsense variants and 7 frameshift variants) or protein-altering variants (n = 10, 9 missense and one in-frame deletion of one amino acid). One variant, c.2653C > T, (p.Arg885*) was identified in two unrelated cases. Among the 20 index cases where parental testing was performed, eight variants were *de novo*, and twelve were inherited from an affected parent. One frameshift variant, c.121del, p.(Asp41Metfs*25), in individual N_13, was mosaic in both peripheral blood (27%) and saliva. According to the ACMG criteria, 22 variants were classified as pathogenic or likely pathogenic (LOF variants and four protein-altering variants), while five protein-altering variants were classified as variants of uncertain significance (VUS). Combining our data with previously published cases, this report includes 41 heterozygous *CTNND2* variants in 40 unrelated cases. 30 were null variants: 16 large deletions, two translocations, five nonsense variants, and seven frameshift variants. In addition, 16 individuals harbor heterozygous protein-altering variants comprising five VUS and six likely pathogenic variants (Fig. 1A, Additional file 1: Table S1).

### Frequency of CTNND2-associated clinical manifestations

#### Intellectual- and speech development

Motor developmental delay was reported in 37 of 41 individuals (90%) and delayed speech and language development in 29 of 42 (69%). The mean age for independent sitting was 10 months (median 12 months; range 6–17 months), and for walking 19 months (median 18; range 9–36 months). The mean age for first spoken words was 19 months (median 22; range 8–60 months). Expressive speech difficulties, including apraxia, delayed speech, and limited sentence formation with the use of simple sentences, were observed in 56% of individuals (13 of 23 individuals). Deficit vocabulary was also present in several adolescent and adult individuals (N_9, N_11, N_18, N_23, N_31, N_54, N_60). In contrast, 44% (10 of 23) of individuals used everyday speech and could form standard sentences. Individual N_59 had dyslexia and was unable to read at 19 years of age, and N_32 could neither read nor write at age 10.

ID or borderline ID was diagnosed in 29 of 39 individuals (74%). ID severity was categorized as moderate to severe in 13%, mild in 26% and borderline in 26%. In addition, three individuals were reported to have learning difficulties without ID. Formal IQ testing was available for 10 individuals aged 5 years or older (mean age 13 years; range 5–40 years), revealing a mean IQ of 70 (range 38–92).

Analysis of developmental delay, ID and speech difficulties showed no evidence of more severe involvement in males compared to females. Interestingly, six individuals with early motor and speech delays demonstrated developmental improvement over time and were reported to catch up on key milestones.

#### Abnormalities of the nervous system

Epilepsy or seizures were reported in 6 of 40 individuals (15%), with five (13%) having a diagnosis of epilepsy. In family F, the father (N_11) reported epilepsy onset at 5 years, and his son (N_9) diagnosed with absence epilepsy at 2 years. N_64 had focal epilepsy and N_16 had focal to bilateral tonic-clonic seizures. One individual (N_30) showed increased seizure susceptibility on EEG without clinical seizures. In individual N_23 epilepsy type was not specified.

Brain magnetic resonance imaging (MRI) was performed in 20 individuals; 13 had normal results and 7 (35%) showed abnormalities, including thin or absent corpus callosum (n = 2), subcortical heterotopia (n = 1), perisylvian polymicrogyria (n = 1), Chiari I malformation (n = 1), diffusion restriction (n = 1) and reduced grey-white matter differentiation (n = 1).

#### Atypical behavior

Autism or autistic traits were documented in 21 of 41 individuals with a formal diagnosis of autism in 14 individuals (34%). ADHD or attention deficit disorder (ADD) was present in 58% (14 of 24) of individuals with LOF variants, but only in 8% (15 of 36) of individuals with protein altering variants, a difference that was statistically significant (p = 0.004 Fisher’s exact test). Atypical behavior was described by attending physicians in 79% (27 of 34) of individuals. Reported features included aggressive behavior (7 of 34; 21%), abnormal emotional behavior (6 of 34; 18%) and impaired social behavior (4 of 34; 11%). In addition, one girl (N_8) had abnormal stereotypical hand movements, and her brother (N_9) showed nervous encopresis.

#### Dysmorphic features

Facial characteristics were variable, with dysmorphic features reported in 30 of 45 individuals (67%) including 20 with LOF variants and 10 with protein-altering variants. The most frequent dysmorphic features involved palpebral fissures, present in 14 of 41 individuals (34%), including upslanting (n = 6), downslanting (n = 5), long (n = 2) and almond shaped palpebral fissures (n = 1). Additional features included low-set ears (n = 3) and craniofacial features in seven individuals, such as plagiocephaly, broad forehead, and retrognathia.

GestaltMatcher analysis of facial photos from 15 *CTNND2* individuals showed that 85% of the intra-cohort similarity distribution overlapped with the range of randomly sampled controls, suggesting that *CTNND2* individuals, as a group, do not exhibit a consistent or distinctive facial gestalt (Fig. 1B, Additional file 3: Fig S2 A). In pairwise similarity analysis (Additional file 3: Fig S2 B), only a subset of individuals, specifically 17 and 17a (siblings), 7, 8, 9, 10 (another family cluster), as well as 43 and 23, formed a visible cluster, with patients 58 and 19 also showing notable similarity. These results indicate that while facial similarities exist among certain individuals, the cohort as a whole does not exhibit a consistent or distinguishable facial pattern, though larger sample sizes may be required to draw firmer conclusions. In summary, both clinical observation and computational analysis suggest that although some *CTNND2* individuals display dysmorphic facial features, no distinct facial gestalt is consistently observed.

#### Additional symptoms

Short stature were reported in two individuals, while all others had normal height. Ophthalmologic features were present in 17 of 45 individuals (38%). Myopia was present in 8 individuals and strabismus in four. Additionaly, discrete muscular hypotonia was observed in four individuals, and hyperextensible joints in six of 39 individuals (15%).

#### Familial CTNND2 Variants and Phenotypic Variability

In twelve of the previously unpublished cases, the *CTNND2* variant was inherited from an affected parent. In addition, the missense variant c.3163C > G, p.(Arg1055Gly) was identified in three siblings, and is presumed to be inherited, although parental samples were not available for testing.

**Figure 2:**
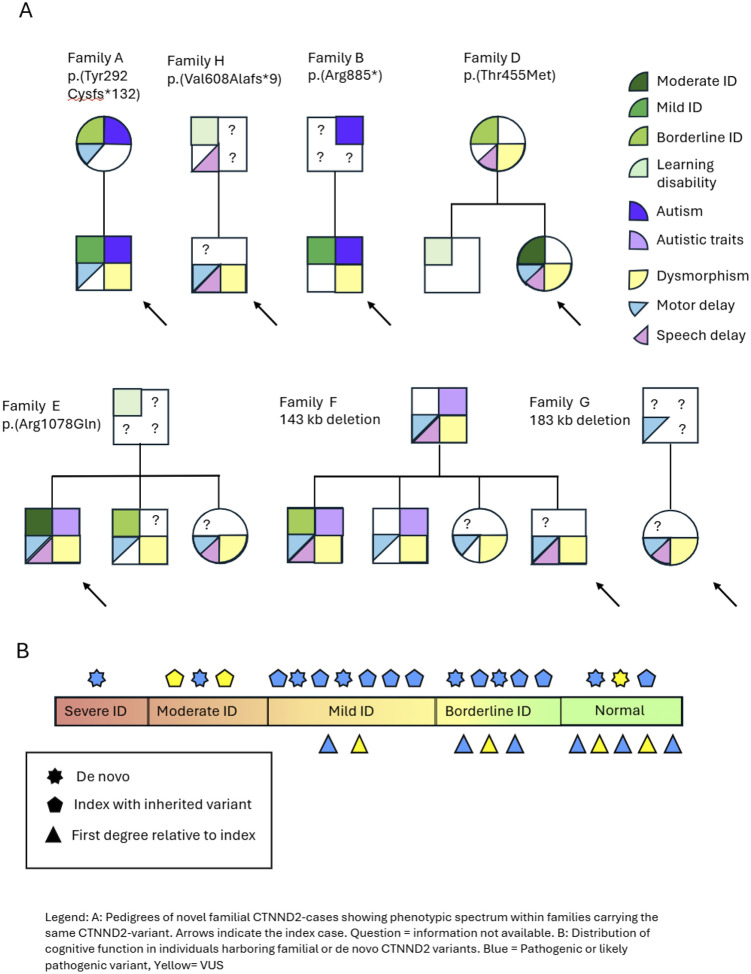
Pedigrees and spectrum in index vs relatives. **A.** Pedigrees of novel familial CTNND2-cases showing phenotypic spectrum within families carrying the same CTNND2-variant. Arrows indicate the index case. Questionmark= information not available. **B.** Distribution of cognitive function in individuals harboring familial or de novo CTNND2 variants. Blue= Pathogenic or likely pathogenic variants, Yellow= VUS.

Clinical manifestations varied significantly among family members carrying the same variant. For example, in families D and E, the index patient had moderate ID while other family members presented with borderline ID, learning disability or no reported ID (Fig. 2). In family F, the degree of language impairment differed between family members: the index individual (N_7) had difficulties forming sentences; his sister (N_8) had normal speech; his brother (N_9) had limited vocabulary and impaired sentence formation, with comprehension and expressive language at the age 12 corresponding to a developmental level of 7.5 years. Another brother (N_10) had a clear articulation disorder, dyslalia and echolalia and a limited vocabulary. Their father (N_11) also had difficulties forming sentences.

In individuals with *de novo CTNND2* variants and index cases, the cognitive spectrum ranged from normal cognition to severe ID. In contrast, none of the family members who harbored the inherited *CTNND2* variant had moderate or severe ID.

#### Loss of CTNND2 disrupts early neurogenesis in 2D models

**Figure 3:**
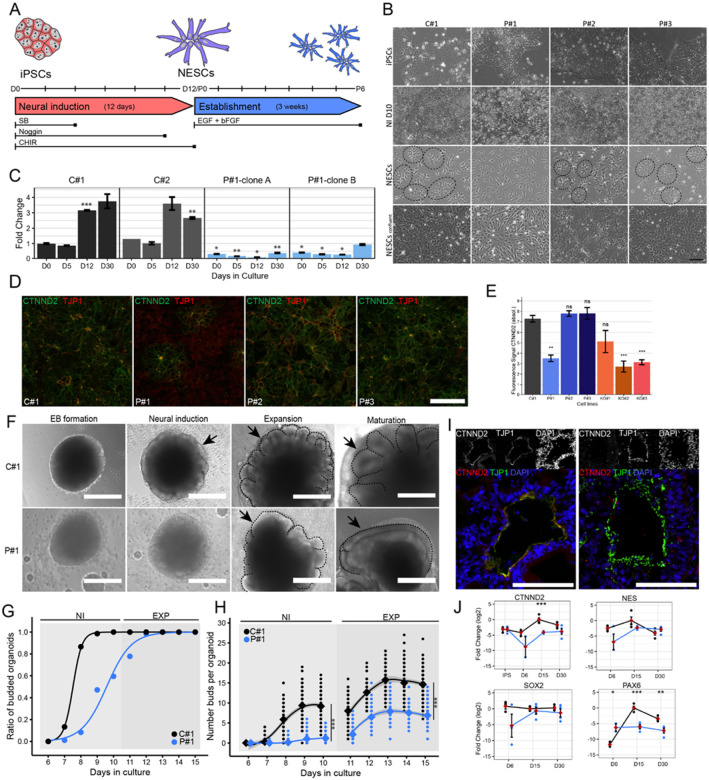
CTNND2-related defects in early neural development. **A.** Experimental setup to establish neuroepithelial stem cells (NESCs) starting with induced-pluripotent stem cells (iPSCs). Neural induction was performed for 12 days using induction medium supplemented with SB (days 1–4), hNoggin (days 1–10), and CHIR (days 1–12). The establishment phase, in NESC medium supplemented with EGF and bFGF, continued for up to six passages (D30). **B.** Bright-field microscopy showed the morphology of the control line (C#1) and three patient-derived cell lines (P#1–3) at iPSC, D10 of neural induction (NI), and NESC culture with low and high confluency per line. Neural rosettes are marked with dashed circles. **C.** The bars represent mRNA expression levels of CTNND2 with standard error of the mean at four distinct time points: D0 (iPSCs), D5, D12 (of the neural induction), and D30 (established NESCs) from control iPSCs and two distinct P#1 iPSC clones. **D.** Immunofluorescent staining of CTNND2 and TJP1 proteins in NESC culture from C#1, KO#1–3, and P#1–3. Scale bar 100 μm. **E.** Quantifications of the fluorescence signal of CTNND2 in the control, knockout and patient NESC lines. Error bars represent error of the mean. *: p<0.05, **: p<0.01, ***: p<0.001. **F.** During EB both patient and control organoid lines showed seemingly healthy structures with translucent peripheral cell layers. The development of neural ectoderm during NI was observed in the P#1 line three days later with a rapid change during the EXP phase. The EXP phase enabled the organoids to enlarge the neural tissue (=neural buds) into the matrigel environment (arrows). **G.** Quantification of the neural bud development of C#1 (black) and P#1 (blue) showed that most C#1 developed organoids by day 8 while P#1 developed slower. **H.** C#1 organoids (black) showed significantly more neural buds per organoid than P#1 (blue) during NI and EXP (multiple regression analysis between the organoid lines, with post hoc two-sided unpaired t-test, ***: p < 0.001). **I.** On day 15, C#1 showed CTNND2 and TJP1 co-localization at the apical side of the early neural tube while P#1 showed no signal for CTNND2. Scale bar 100 μm. **J.** Gene expression of C#1 (black) showed a higher expression of CTNND2 compared to P#1 (blue). Additionally, early neural markers SOX2, NESTIN and PAX6 are active during NI in C#1 but show less fluctuating and lower expression in P#1. Error bars represent error of the mean. Two-sided unpaired t-test, *: p<0.05, **: p<0.01, ***: p<0.001.

To investigate the role of *CTNND2* in the early human brain development, iPSCs from three affected individuals (two females: P#1 (N_48) and P#2 (N_49), and an unrelated male: P#3 (N_7)), three knockout cell lines (KO#1 heterozygous, KO#2 homozygous, KO#3 homozygous) (Additional File 3: Fig S1 A, B), and three controls (C#1, C#2, and C#3) (Additional File 1: Table S2) were differentiated into NESCs as described previously (Calvo-Garrido et al., 2021; Falk et al., 2012; Shahsavani et al., 2017) (Fig. 3A, B and Additional File 3: Fig S1 C). During the 12-day neural induction phase, cells transition from pluripotent stem cells to neuroepithelial stem cells, accompanied by increased proliferation, nucleal shrinking, cytoplasmic expansion and elongation into a mermaid-like shape, ultimately forming neural rosette patterns (Falk et al., 2012).

During neural induction, P#1 cells consistently showed reduced proliferation and disorganized, scattered growth, failing to form stable neural rosettes or maintain expandable NESC lines unlike P#2, P#3, knockout, and control lines (Fig. 3B, Additional File 3: Fig S1 C). These findings suggest impaired self-organizing properties of P#1 NESCs.

We next examined *CTNND2* expression during the neural induction. In control lines (C#1 and C#2), qPCR analysis revealed robust upregulation of *CTNND2* on day 12, which persisted in established NESCs (day 30) (Fig. 3C). In contrast, *CTNND2* expression averaged across day 12 and day 30 for all samples was 6.6-fold lower in two iPSC-derived clones from P#1 (range 0.07–0.97, mean 0.50, p < 0.05) compared to controls (range 2.60–4.21, mean 3.2, p < 0.01) (Fig. 3C). Consistent with these findings, quantitative analysis of immunolabeled NESCs on day 30 showed that P#1 cells had approximately 50% lower CTNND2 protein expression compared to P#2, P#3, and C#1 (two-sided unpaired t-test, Bonferroni corrected, p = 0.0007) (Fig. 3D–E, Additional File 3: Fig S1 D).

#### Loss of CTNND2 impairs development of cerebral organoids

To further investigate the developmental impact of *CTNND2* variants, cerebral organoids were generated from P#1 and C#1 iPSCs (Lancaster et al., 2013). The 30-day culture process was divided into four phases: embryoid body formation (EB; days 1–7), neural induction (NI; days 8–11), expansion (EXP; days 12–15), and maturation (MAT; days 16–30) (Additional File 3: Fig S3 A).

During the EB stage, P#1 iPSCs formed EBs 21%-24% more slowly than C#1 (two-sided unpaired t-test, p < 0.001, day 3; p < 0.001, day 12), resulting in an overall growth delay of 1–3 days by day 30 (Additional File 3: Fig S3 B-C). Increased cell debris was observed in P#1 during the EB phase (Fig. 3F), and greater variability in organoid area was noted at day 6 (Additional File 3: Fig S3 B). In the NI phase, the C#1 organoids formed as expected with a smooth periphery while in P#1, the process was delayed by three days (Fig. 3F). The P#1 organoids then developed irregular and less circular enlargements during the EXP phase compared to the C#1 organoids (Fig. 3F, Additional File 3: Fig S3 C). In the MAT phase, the C#1 organoids developed extended yet compact buds and P#1 organoids formed less compact and elongated buds (Fig. 3F).

To quantify neurogenesis, we measured neural bud formation, a key indicator of neural tissue organization. Both lines showed a significant increase in neural bud formation over time (p < 0.001), consistent with normal developmental progression. However, P#1 organoids demonstrated a 3.5-fold reduction in overall neural bud formation compared to C#1 (p < 0.001, Fig. 3G). Specifically, P#1 organoids showed an 86% decrease in the number of buds per organoid during the NI phase (p < 0.001) and a 52% reduction during the MAT phase (p < 0.001, Fig. 3H). These results indicate a substantial deficit in neural bud development in P#1 organoids.

To explore the molecular basis of these developmental phenotypes, we examined key neural markers. By day 15, both organoid lines formed neural tube-like structures positive for the apical marker TJP1 (Fig. 3I). However, co-localization of CTNND2 and TJP1 in P#1 organoids, observed in C#1, was absent in P#1 organoids (Fig. 3I). Both lines showed radial expression of NESTIN and SOX2, but CTNND2 expression was consistently reduced in P#1 organoids across all time points and lacked the day 15 upregulation seen in C#1 (Fig. 3J, Additional File 3: Fig S3 D). Early neurodevelopmental markers (SOX2, NESTIN, PAX6) increased during neurogenesis in C#1 organoids and declined by day 30, while P#1 organoids showed lower overall expression and failed to downregulate these markers at later stages (Fig. 3J). Proliferation, assessed by MKI67 expression, was comparable between (Additional File 3: Fig S3 E). However, neuronal maturation markers (TUBB3, DCX, MAP2) were significantly lower in P#1 organoids at day 30, indicating impaired neuronal differentiation (Additional File 3: Fig S3 F).

#### Transcriptome analysis highlights WNT dysregulation in CTNND2-related neurogenesis

To examine molecular changes during neural induction, we performed RNA-seq on iPSC-derived neural stem cells from affected individuals, knockout lines, and controls at two stages: day 12 of neural induction (passage 0: P0) and in established NESCs at passage 6 (P6), generating a comprehensive transcriptomic dataset for early neurogenesis.

**Figure 4:**
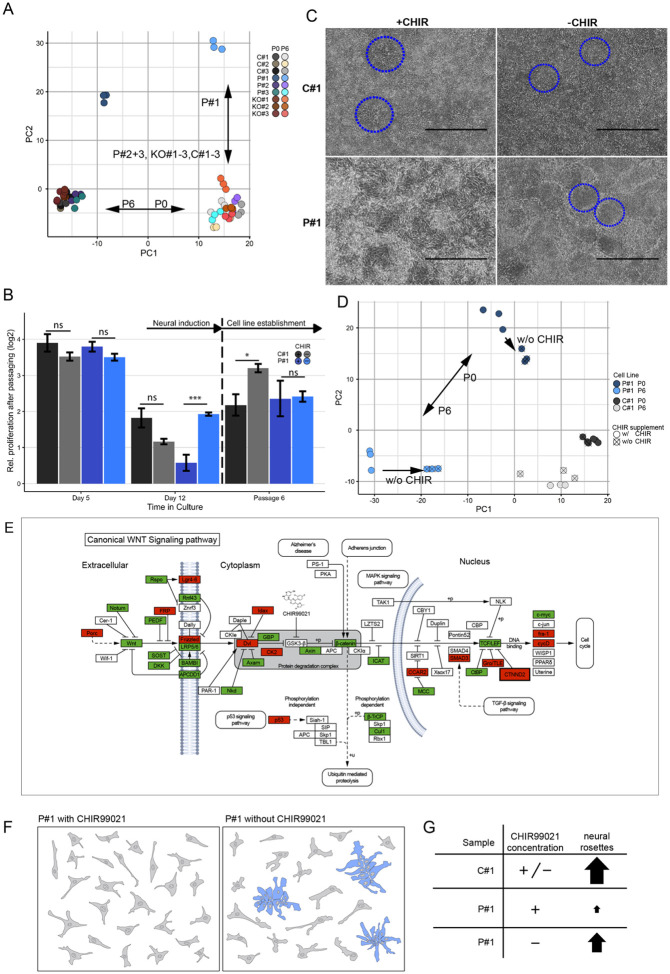
CHIR-dependent rescue of early neural defects in P#1 Cells. A. Principal component analysis of transcriptome data of C#1–3, KO#1–3 and P#1–3 NESCs from two different time points: P0 (Day12 of neural induction) and P6 (NESCs passage 6). B. Proliferation analysis to assess the effect of CHIR on C#1 and P#1 during neural induction and cell line establishment. Error bars represent the standard error of the mean. Statistical analysis with two-sided unpaired t-tests. *: p<0.05, **: p<0.01, ***: p<0.001. C. Brightfield microscopy images of CHIR removal from neural induction on day 12/P0. The control line C#1 developed neural rosettes (blue circles) with (+) and without (−) CHIR, while the P#1 lacked those and only developed them when CHIR was excluded from the neural induction (right). D. Principal component analysis of transcriptome data from two different conditions (−CHIR and +CHIR) revealed a positive shift of patient cells in the −CHIR condition towards control cells. E. Differentially expressed genes from P#1 NESCs, P0/D12 involved in the canonical WNT pathway were annotated for upregulation (green) and downregulation (red) compared to control cells (modified from the Kyoto Encyclopedia of Genes and Genomes, KEGG. CHIR inhibits GSK3-β leading to unphosphorylated free β-catenin that migrates into the nucleus, binds to TCF/LEF and thus activates gene expression. Arrows: activation, flat arrows: inhibition, dotted arrows: external pathway entries/exits, +p: phosphorylation, +u: ubiquitination. F, G. P#1 cells yield mostly single irregular cells (left) when neurally induced in the presence of CHIR (left). Without CHIR, neural induction partially rescues the phenotype, revealing neural rosettes (blue) in P#1 NESCs (right). C#1 NESCs develop neural rosettes independently of CHIR concentration.

The presence of the respective *CTNND2* variants was first confirmed in the RNA-seq data across all lines. PCA showed distinct clustering of technical replicates within each sample, validated by the Kruskal-Wallis test (p < 0.001) (Fig. 4A). Tight clustering was observed among P#2, P#3, KO#1–3, and C#1–3 at both time points, with no significant differences in transcriptomic profiles between lines (Kruskal-Wallis, n.s.), indicating overall similarity. In contrast, comparison between time points revealed a significant effect of developmental stage on gene expression profiles (p < 0.001), reflecting transcriptional changes during neural differentiation.

We next evaluated *CTNND2* expression at the two stages (Additional File 3: Fig S4 A). Control lines showed consistent expression patterns, with higher levels at P0/D12 that decreased to ~ 60% at P6, reflecting normal downregulation. KO#1 and KO#2 displayed consistently lower expression, about 50% of controls at both time points. In contrast, KO#3 initially showed control-like expression at P0/D12 but resembled other knockout lines by P6. The patient derived lines displayed greater variability: P#1 showed minimal *CTNND2* expression at both time points, while P#2 displayed intermediate expression levels between P#1 and P#3.

Motivated by the distinct characteristics of P#1 cells during neural induction and the PCA results, we focused our analysis on day 12/P0, comparing P#1 with controls (C#1, C#2, C#3). Although P#1 cells showed a similar time-dependent gene expression shift as P#2 and P#3, the PCA revealed a clear separation of P#1, primarily driven by the second principal component. Using an FDR of 1%, we identified 5,381 differentially expressed protein-coding genes (DEGs) between P#1 and control cell lines. Of these, 2,544 genes were downregulated and 2,837 were upregulated in P#1 NESCs (Additional File 3: Fig S4 B).

To explore the biological processes underlying the observed transcriptional changes, we performed pathway enrichment analysis. Among the top five enriched pathways, were three key signaling pathways (WNT 1.64x; Cadherin 1.79x and Integrin 1.6x; Fisher’s Exact test) (Additional File 3: Fig S4 C). Specifically, within the 309 WNT associated genes in the PANTHER database, 128 were differentially expressed in P#1 cells at P0, with 82 genes (64%) upregulated and 46 genes (36%) downregulated (Additional File 1: Table S5).

#### WNT modulation rescue

To explore whether WNT pathway dysregulation contributed to the abnormalities observed in P#1 cells during neural induction, we modified the neural induction protocol by removing the WNT activator CHIR99021 (here after called CHIR) from the culture media (Fig. 4F, Additional File 3: Fig S5). Neural induction was performed under two conditions: one with CHIR (+ CHIR, 3.3 μM) and one without CHIR (−CHIR).

During the initial stage of neural induction, both P#1 and control cells proliferated extensively, regardless of CHIR treatment (Fig. 4B, day 5). However, differences in proliferation emerged after replating on day 5 and continued to day 12, coinciding with increased *CTNND2* expression. In the absence of CHIR, P#1 cells doubled their proliferation rate, reaching levels comparable to C#1 cells grown with CHIR (Fig. 4B). In contrast, with CHIR present, P#1 cells failed to match the proliferation rates of C#1. Furthermore, neural rosette patterns formed in the −CHIR P#1 culture condition, resembling those observed in C#1 cultures under both conditions (Fig. 4C).

Transcriptome analysis of the two conditions (−CHIR and + CHIR) supported the observed rescue phenotype. PCA showed clear separation between P#1 and C#1 cells, as well as between the treatment conditions (Fig. 4D). Notably, removal of CHIR led to a significant transcriptomic shift in P#1 cells toward the control profile (Kruskal-Wallis, p = 0.004) (Fig. 4D). In contrast, CHIR removal had no significant effect on the transcriptome of C#1 cells (p = 0.109).

Transcriptomic profiles of P#1 and C#1 cultured without CHIR were compared to their respective + CHIR conditions, serving as cell line-specific baselines. This approach allowed us to assess the effect of CHIR on each cell line while accounting for unrelated cell line-specific gene expression differences. In C#1, 4,578 DEGs were identified, and in P#1, 3,433 DEGs were found, with roughly equal proportions of upregulated and downregulated genes (Additional File 3: Fig S4 D). Focusing on WNT pathway-associated DEGs in this comparison, 68% of WNT genes were upregulated and 32% downregulated in C#1 (Additional File 3: Fig S4 E). P#1 showed a similar distribution, with 64% upregulated and 36% downregulated WNT genes. However, the specific DEGs were not entirely shared between the two cell lines. To address this, we filtered for overlapping genes and compared their expression profiles side by side (Additional File 3: Fig S4 F). This analysis revealed 42 shared genes, of which 19 (*CDH23*, *CDHR1*, *PRKCA*, *CTNNA3*, *CSNK2A1*, *FZD7*, *MYCL*, *PLCB1*, *SIAH2*, *NKD1*, *PCDH18*, *ACTC1*, *ACTB*, *PCDHA4*, *PCDHA12*, *EDN1*, *WNT5A*, *GNG3*, *GNG10*) displayed opposite expression patterns, with increased expression in P#1 and decreased expression in C#1, or vice versa.

## DISCUSSION

In this comprehensive study on CTNND2-related neurodevelopmental disorders, we combined detailed clinical phenotyping with functional studies in patient-derived iPSC models. Our cohort of 57 individuals, including 42 newly described and 15 previously reported cases, defines a core clinical presentation of intellectual disability and behavioral abnormalities, alongside a variable spectrum of neurological manifestations and dysmorphic features. Functional neural models further support a role for CTNND2 in early neurogenesis and neuronal maturation, highlighting its involvement in regulating WNT signaling pathways during early neural development.

This study presents the largest clinical cohort of individuals with *CTNND2* variants reported to date, providing a comprehensive view of the associated neurodevelopmental disorder. Analysis of 38 unrelated cases revealed a broad phenotypic spectrum, ranging from very mildly affected individuals to those with moderate or severe intellectual disability. This variable expressivity was observed even within the same family. Apart from a higher prevalence of ADD/ADHD and atypical behavior in individuals with LOF-variants, no other statistically significant genotype-phenotype correlation was observed between variant types and symptom severity did not differ by gender. A trend toward a more severe ID in *de novo* and index cases was noted (Fig. 2), though this may reflect ascertainment bias, as individuals with more severe or atypical symptoms are more likely to be referred for genetic testing. Milder or subclinical cases may remain underrecognized, skewing the observed distribution of phenotypic severity. Facial analysis by GestaltMatcher (Hsieh et al., 2022) did not identify a consistent facial gestalt (Fig. 1B), though some individuals showed overlapping features (Additional file 3: Fig S2). This further emphasizes the variable expressivity of *CTNND2*-related disorders and the challenge of clinical diagnosis. These disorders fall within the broader group of neurodevelopmental conditions characterized by intellectual disability, behavioral abnormalities, and variable neurological and cognitive features. The clinical heterogeneity reflects the complex interplay of *CTNND2* haploinsufficiency with additional genetic, environmental, or epigenetic modifiers, highlighting the importance of integrating genomic data with careful clinical assessment.

To better understand the cellular basis of the variable clinical presentations, we studied early neurogenesis in iPSC-derived models from three individuals with heterozygous *CTNND2* loss. Notably, only one line (P#1) showed impaired neural rosette formation and failed to establish stable NESCs, while lines from her mother (P#2) and an unrelated individual (P#3) differentiated as control lines. This variability, despite similar CTNND2 disruption, suggests additional factors, such as genetic background, epigenetic modifications, or stochastic variation, modulate phenotypic expression. These observations align with findings from model organisms, where the expressivity and penetrance of identical mutations are shaped by complex genetic interactions beyond the primary disease-causing variant (Kammenga, 2017). Supporting this, CRISPR-Cas9 knockout lines also showed normal rosette formation, indicating that *CTNND2* haploinsufficiency alone is insufficient to explain the neural defects. Given these findings, we extended our investigation to cerebral organoids, a 3D model of early brain development that captures key processes such as proliferation and differentiation (Andrews & Kriegstein, 2022; Paulsen et al., 2022). Organoids derived from P#1 recapitulated the neural induction defects observed in 2D cultures, showing delayed neurogenesis, abnormal neural bud formation, and reduced expression of early brain markers. These abnormalities likely reflect impaired cell–cell interactions and disrupted tight junction regulation, resulting in defective neural rosette formation and disorganized tissue growth.

To further investigate the molecular mechanisms underlying these neurodevelopmental defects, we performed transcriptomic analysis of the 2D neural stem cell model. RNA-seq of P#1-derived NESCs revealed significant dysregulation of WNT signaling a pathway critical for early neural development and rosette formation. Specifically, we observed increased expression of key downstream effectors, including β-catenin and TCF/LEF, alongside reduced expression of upstream components such as Frizzled receptors, DVL, and CTNND2 itself (Fig. 4E). This imbalance suggests a shift in WNT pathway dynamics, potentially contributing to the impaired neurogenesis observed in P#1. While these findings demonstrate clear transcriptional changes, how they translate into altered protein levels and pathway activity remains unclear. Future proteomic studies will be essential to define the functional consequences of these gene expression alterations and to better understand how CTNND2 loss disrupts WNT pathway regulation in human neural progenitors.

Neural rosette formation is a critical morphogenetic process, the first essential structure in neurogenesis, tightly regulated *in vivo* to maintain the proper development of neural stem cells (Malchenko et al., 2014). Previous work has implicated WNT signaling as a key regulator of this process (Elkabetz et al., 2008). Consistent with these findings, our data suggest that loss of CTNND2 disrupts WNT pathway balance, impairing rosette formation and neural progenitor organization. The inability of neurally induced P#1 iPSCs to form neural rosettes and maintain stable NESC lines highlight the essential role of *CTNND2* in this early developmental stage.

To further explore whether WNT dysregulation underlies the observed defects, we tested whether modulating WNT signaling could restore normal neural induction in P#1 cells. Remarkably, removing the WNT activator CHIR from the neural induction protocol partially rescued both proliferation and neural rosette formation. In the absence of CHIR, P#1 cells exhibited improved growth and more organized radial clusters, resembling the morphology of control NESCs (Fig. 4F, G). In contrast, when WNT signaling was activated by CHIR, P#1 NESCs remained disorganized and scattered, failing to form rosettes. The vulnerability of P#1 cells to WNT signaling modulation indicates a possible fine-tuning requirement for precise WNT levels during neural induction. These findings suggest that WNT signaling contributes to the neurodevelopmental abnormalities in P#1 cells, and that precise regulation of WNT activity may be critical during early neural development.

Both the clinical presentation of the 53 individuals reported here with *CTNND2*-related neurodevelopmental disorders and the *in vitro* model of three patient-derived and three CRISPR-Cas9 generated neural cell lines show a high degree of variability. The CRISPR-Cas9-generated knockout lines, despite harboring *CTNND2*-disruptive alleles, showed normal growth and neural rosette formation, resembling P#2, P#3, and controls. While the induced indels reduced *CTNND2* transcript levels, RNA-seq still detected residual expression, likely due to alternative downstream start codons. These transcripts may be non-functional or partially functional, as the coding sequence was disrupted. Such incomplete gene disruption, referred to as “knockout escape”, has been described previously both in zebrafish and human iPSC models (Anderson et al., 2017; Sebastian et al., 2023) and reflects the limitations of CRISPR-based models in fully capturing the complexity of human disease (Wang et al., 2024). In contrast, patient-derived lines, with their intact genetic complexity, may provide a more accurate representation of disease mechanism. However, even though we included three patient lines in the current study, phenotypic abnormalities were only detected in one of them. This variability is not unique to CTNND2. Similar heterogeneity has been reported in iPSC-based models of other neurodevelopmental disorders. For example, in 16p11.2 deletion models, ventral forebrain organoids exhibited significantly greater variation in size and rosette formation, with some cultures producing very few rosettes (Fetit et al., 2023). Similarly, in ASD iPSC models, some patient-derived lines show pronounced neural rosette abnormalities, whereas others appear unaffected (Adhya et al., 2021). This observed variations in the neural stem cells underscore the need for larger sample sizes to fully understand the complexities of neurodevelopmental disorders and to capture the complete spectrum of disease-relevant phenotypes, especially those with variable penetrance.

Together with previous observations, these findings highlight the need for caution when interpreting gene disruption models, as knockout alone may not fully capture the complexity of human disease phenotypes. While our cellular studies of one individual (P#1) revealed a striking WNT-driven neurogenesis defect, the broader clinical cohort demonstrated that *CTNND2* haploinsufficiency leads to a highly variable neurodevelopmental phenotype, ranging from mild to severe. This variability underscores the influence of additional genetic and environmental modifiers on disease severity. Our findings place *CTNND2* alongside other WNT-regulating genes such as *APC*, *CHD8*, and *CTNNB1*, whose disruption similarly causes variable neurodevelopmental disorders (de Ligt et al., 2012; Krumm et al., 2015; Nakagawa et al., 2017). Collectively, these conditions define a growing class of WNTopathies, highlighting the critical role of finely tuned WNT signaling in early brain development (Pascual et al., 2025).

## CONCLUSIONS

This study defines *CTNND2*-related neurodevelopmental disorders as a clinically variable condition characterized by intellectual disability and behavioral abnormalities. Through international collaboration, we present genotype and phenotype data from 57 individuals, the largest cohort to date. Functional studies using patient-derived iPSC models demonstrate that *CTNND2* is essential for neural rosette formation and early neurogenesis, acting through WNT signaling. Notably, only one patient-derived line exhibited pronounced defects, highlighting the impact of genetic background and potential compensatory mechanisms. Together, our findings identify *CTNND2*-related disease as a neurodevelopmental WNTopathy and emphasize the importance of precise WNT signaling during neural induction.

## Supplementary Material

This is a list of supplementary files associated with this preprint. Click to download.

- AdditionalFile1TablesS15.xlsx
- AdditionalFile2Supplementarymethods.docx
- Additionalfile3FigS15.docx

## Figures and Tables

**Table 1:** Summary of clinical features of *CTNND2*-related neurodevelopmental disorder. HPO: Human Phenotype Ontology; LOF: Loss of Function; ADHD: Attention-Deficit/Hyperactivity Disorder; ADD: Attention-Deficit Disorder.

Phenotypical manifestation	HPO	LOF n = 41, 72%		Protein altering n = 16, 28%		Total n = 57	
**Intellectual, social and behavioral development**							
Developmental Delay	0001263	25/28	89%	12/13	92%	37/41	90%
Delayed speech and language development	0000750	17/28	61%	12/14	86%	29/42	69%
Intellectual disability (including borderline ID)	0001249	19/26	73%	10/13	77%	29/39	74%
Intellectual disability, borderline	0006889	9/26	35%	1/13	8%	10/39	26%
Intellectual disability, mild	0001256	8/26	31%	2/13	15%	10/39	26%
Intellectual disability, moderate	0002342	0/26	0%	4/13	31%	4/39	10%
Intellectual disability, severe	0010864	0/26	0%	1/13	8%	1/39	3%
**Autism**	0000717	10/28	36%	4/13	31%	14/41	34%
**Atypical behavior**	0000708	20/23	87%	7/11	64%	27/34	79%
Aggressive behavior	0000718	6/23	26%	1/11	9%	7/34	21%
Abnormal emotional behavior	0100851	4/23	17%	2/11	18%	6/34	18%
Abnormal social behavior	0012433	2/23	9%	2/11	18%	4/34	11%
**ADHD/ADD**	0000736/0007018	14/24	58%	1/12	8%	15/36	42%
**Epilepsy**	0001250	4/25	16%	1/15	7%	5/40	13%
EEG abnormality	0002353	5/8	63%	1/4	25%	6/12	50%
Brain imaging abnormality	0410263	4/13	31%	3/7	43%	7/20	35%
**Facial dysmorphism and body abnormalities Intellectual, social and behavioral development**	0001999	20/32	63%	10/13	77%	30/45	67%
Abnormal shape, size, or slanting of palpebral fissure	0200005,0200006,0200007	12/29	41%	2/12	17%	14/41	34%
Hyperextensible joint	0001282	5/23	22%	1/16	6%	6/39	15%
Myopia	0000545	8/32	25%	0/12	0%	8/44	18%

## Data Availability

RNA-Seq data were deposited in the European Genome-phenome Archive (EGA) under the accession number EGA50000000309 and is available upon request.

## Abbreviations

ADD: Attention-Deficit Disorder
ADHD: Attention-Deficit/Hyperactivity Disorder
iPSCs: induced-pluripotent stem cells
LOF: Loss of Function
NDD: Neurodevelopmental disorders
NESCs: Neuroepithelial stem cells
VUS: variants of uncertain significance
